# Development and validation of care stress management scale in family caregivers for people with Alzheimer: a sequential-exploratory mixed-method study

**DOI:** 10.1186/s12877-023-03785-6

**Published:** 2023-02-07

**Authors:** Hamid Sharif Nia, Lida Hosseini, Mansoureh Ashghali Farahani, Erika Sivarajan Froelicher

**Affiliations:** 1 Traditional and Complementary Medicine Research Center, Addiction Institute, Mazandaran University of Medical Sciences, Sari, Iran; 2 School of Nursing and Midwifery, Iran University of Medical Sciences, Tehran, Iran; 3 Centers for Nursing Care Research, School of Nursing and Midwifery, Iran University of Medical Sciences, Tehran, Iran; 4 Department of Physiological Nursing, Department of Epidemiology & Biostatistics, Schools of Nursing and Medicine, University of California San Francisco, San Francisco, CA USA

**Keywords:** Stress management, Validity, Reliability, Psychometrics properties, Alzheimer

## Abstract

**Background:**

Caring for a person with Alzheimer’s disease is stressful for caregivers. So that, considering all the emotional and financial costs imposed on the families of Alzheimer’s patients, stress from caring is an issue that cannot be ignored and plans need to be developed to help these caregivers to manage the care properly. The current study was designed to develop a valid and reliable care stress management scale for family caregivers of patients with Alzheimer’s.

**Methods:**

This study is a methodological study with a sequential-exploratory mixed-method approach that was performed in two-phase: develop the caring stress management scale and evaluate the psychometric properties of the scale. In the first phase, 14 semi-structured face-to-face interviews were performed with family caregivers of patients with Alzheimer’s. The interviews were transcribed immediately and an item pool with 275 items was prepared. After removing the duplicate or overlapping code, the initial format of the caring stress management scale (CSMS) was designed. In the second step, the items of the CSMS were evaluated using face and content validity. After that, the construct validity was evaluated using exploratory factor analysis, confirmatory factor analysis, and convergent and divergent validity respectively. Finally, the reliability was assessed by stability and internal consistency. The sample size was 435 and data was gathered via an online form questionnaire.

**Results:**

This study designed the CSMS with two factors including emotional-focused coping (4 items) and problem-focused coping (4 items) that explained 51.00% of the total variance. The results of the confirmatory factor analysis showed a good model fit. Furthermore, the internal consistency and stability of this scale were acceptable.

**Conclusion:**

The results showed that the care stress management scale has two factors in Iranian family caregivers and it is valid and reliable and can be used by therapists and researchers.

## Background

As the elderly population is increasing globally, the number of people with dementia is also increasing [1]. So that, 46.8 million people had dementia in 2015 in the world and the number of these patients is expected to almost triple by 2050 [2]. Aging of the population is often seen as a global public health matter, but it is also widely recognized as a public health challenge in high-income countries [3]. This issue has been turned into the greatest healthcare challenge due to its impacts on quality of life and epidemiological, economic, and social impacts [4].

Dementia is a neurologic syndrome that affects memory and cognitive function and is characterized by impairment in cognitive, language function, activities of daily living, and the ability to judge [1]. Alzheimer’s disease (AD) is the most common type of dementia that accounts for 50–70% of all dementia cases [5]. Since AD affects people’s cognitive, functional, and emotional abilities, these patients become dependent on others for their daily needs, and this dependence increases over time [6].

Before institutional care, family caregivers play the most important role in providing care to these patients so that approximately 83% of the care required by these patients is provided by their families [7]. Caring for a person with dementia is a difficult and complex task for family members because they must provide the patient with personal care, housekeeping, medication, processing of financial transaction, and other activities; and as the disease progresses, these tasks become heavier [8].

Due to the long-term and progressive nature of AD, taking care of an older person with Alzheimer’s becomes a 24-hour responsibility. The caregiver may neglect to take care of themselves and exposes them to physical, mental, and financial burdens [9]. This burden may be one of the factors that cause chronic stress in caregivers [10]. This chronic care stress affects various aspects of caregivers’ physical and psychological health [11]. For example, numerous studies have shown that family caregivers suffer from many psychological effects including depression, stress, and strain [1, 12] and they experience at least one type of depressive disorder while caring for their family member [13]. Additionally, prolonged exposure to chronic stress increases the risk of illnesses such as cardiovascular disease and hypertension in these caregivers [11]. As a result of the conditions resulting from caregiving, these people are more likely than non-caregivers to need medical services, such as physician visits, medication, or even hospitalization [14]. Hence, some researchers consider that the stress of caring for a person with Alzheimer’s is far greater than the stress of caring for a person with other illnesses [15]. Therefore, considering all the emotional and financial costs imposed on the families of Alzheimer’s patients, caring stress is an issue that cannot be ignored and plans to help these people to manage the stress and develop skills to cope with the stress [16].

Coping is a process-oriented ability that efforts a person’s way of manage external and internal demands in stressful situations. This occurs by changing cognition and behavior constantly [17]. Coping includes cognitive and behavioral abilities which control internal and external factors causing stress [18]. Based on the literature, there are many ways of coping that are considered as two categories: (1) problem-focused coping and (2) emotion-focused coping [19–21]. Problem-focused coping includes all the active efforts to manage stressful situations and alter a troubled person-environment relationship to modify or eliminate the sources of stress via individual behavior. Emotion-focused coping includes all the regulative efforts to diminish the emotional consequences of stressful events. It is noteworthy that coping strategies depend on the type of stressors and they can changed over time and in various contexts. Also, the experience of individuals in stressful situations is effective on the coping strategies used [17].

About caring from persons with Alzheimer’s, despite the negative effects of the care, it should be noted that how caregivers evaluate these stressors is an important factor in the effects of stress on their coping strategy. Studies have shown that caregivers have shown a different perception of their role [6]. Some people see it as a stressful situation and are experiencing many physical and psychological problems such as depression, stress, and mental pressure [12], while others consider it an opportunity and gain positive experiences including the feeling of satisfaction, skillfulness and capability, improvement of the quality of relationships with patients, and self-efficacy [22]. The experiences that caregivers can have from care depend on their ability to manage care stress and their ability to cope with that situation [23]. This ability is influenced by various factors, including factors of socio-economic status and caregiver resources [23]. Therefore, it is important to identify the ability of caregivers to manage stress to reduce the burden of care and the resulting physical and psychological effects.

To our knowledge, most studies designed to date have focused on describing caregiver burdens and distress, care challenges, and side effects of these burdens and stress. Yet, there is a lack of an appropriate scales to identify the caregiver’s ability to manage this stress. For example, the Neuropsychiatric Inventory Caregiver Distress Scale (NPI-D) was developed to assess the caregiver distress related to the Neuropsychiatric symptom in persons with dementia assessed by Neuropsychiatric Inventory (NPI) [24]. When using this scale, the frequency and severity of each symptom domain of the NPI is rated, then the emotional or psychological distress experienced in relation to that symptom by caregivers is rated on a 6-point scale: 0 (Not at all distressing) to 5 (Very Severely or Extremely distressing). The total score of caregiver distress (NPI-distress) is obtained by the sum of NPI-D scores across the 12 NPI domains [24, 25]. Furthermore, Zarit’s caregiver burden interview (ZBI) is another scale that is used to measure of burden experienced by family members of caregivers of community-residing impaired elders. It includes 22 questions that measure the caregiver’s health, psychological well-being, social life, finances, and the relationship between the caregiver and patient graded on a scale from 0 to 4 [26].

For this purpose, the present study was designed to evaluate the psychometric properties of the care stress management scale in family caregivers of patients with Alzheimer’s disease.

## Methods

### Design

This study used a sequential-exploratory mixed-method design. It was performed in family caregivers of patients with AD in two-phase to design and validate a care stress management scale in family caregivers of Alzheimer’s patients: 1) item generation via interview, and 2) psychometric assessment of the final scale obtained from the item generation phase.

### Item generation

To clarify and explain the concept of the care stress management in family caregivers of patients with AD, identifying related structures, and producing an item pool, fourteen semi-structured face-to-face interviews were performed with family caregivers of AD’s patients from November 2020 to February 2021. These participants were selected via purposeful and snowball sampling with maximum variety. Each interview took between 30 and 90 minutes. The interviews used the following questions:

1. Please describe a day from morning to a night spent caring for your patient?
2. What do you do to deal with care-related problems?
3. What do you do when you are stressed?
4. How do you control the stressful situation of caring for your patient?

In addition, probing questions such as: “Can you explain more about this?”, “Can you give an example?”, “When you say …, What do you mean?” were used during the interview. Using these questions, we wanted to understand how these people manage their stress while caring. All of the interviews were recorded and at the end of each interview, the interview was transcribed immediately and was analyzed with a directed content analysis method using MAXQDA software Ver.10. In this step, 275 initial codes were extracted that were classified. Then based on extracted codes, an item pool with 275 items was prepared. Researchers have reviewed the items several times. Duplicated or overlapped items were removed. Finally, the initial format of the care stress management scale (CSMS) was designed with 18 items with a five-point Likert response (1 = never, 2 = rarely, 3 = sometimes, 4 = often, 5 = always).

### Item reduction

In this stage, the psychometric property of CSMS were assessed using the face, content, and construct validity, as well reliability.

### Face validity

Two qualitative and quantitative approaches were used to assess face validity. Qualitative face validity was performed by asking 10 family caregivers to examine items in terms of the level of difficulty, relevancy, or ambiguity in answering. The items were edited by the research team based on participant recommendations. Quantitative face validity was assessed by calculating the impact score of each item. From the same 10 family caregivers were asked to assess the suitability of each item by following answers: “5= it is completely suitable, 4= it is suitable, 3= it is almost suitable, 2= it is a little suitable, 1= it is not suitable at all.” The impact score was calculated using the formula the impact score = frequency (%) × suitability. The impact score > 1.5 considered acceptable [27]. Accordingly, all items were accepted at this stage.

### Content validity

Two approaches qualitative and quantitative were used to evaluate of content validity of CSMS. The qualitative content validity was assessed by asking 12 experts in nursing, psychology, and instrument development to evaluate the items in terms of grammar, wording, item allocation, and scaling. Some items of the scale were modified according to recommendations. The quantitative content validity of the scale was assessed by calculating the content validity ratio (CVR) and modified kappa coefficient (K). For evaluating CVR, from the same 12 experts was asked to assess the essentiality of items by following answers: 1 = not essential, 2 = useful but not essential, 3 = essential. Based on the Lawshe formula (1975), an acceptable amount of CVR with 12 experts is 0.56 [28]. Four items were removed (CVR < 0.56). After that modified Kappa (K) was evaluated by asking the 11 different experts to assess the relevancy of each item by the dichotomous response: 1 = relevant, 0 = irrelevant. The score of K > 0.75 was considered excellent and the score 0.60 to 0.74 was considered good [29]. One item was removed and totally the items of CSMS were reduced to 13 items.

### Item analysis

Before entering the construct validity step, item analysis was performed to identify possible problems of items by calculating the corrected item-total correlation. For this purpose, the online form of the questionnaire was designed and its link was sent to 32 family caregivers through WhatsApp and Telegram (mean age 52.02 ± 13.91). The correlation coefficient between cases less than 0.32 was considered as the criterion for removing items [29]. Two items were removed according to corrected item-total correlation of ≤0.32 and the total items of CSMS reduced to 11 items.

### Construct validity

#### Participations and samples

The target population was Iranian family caregivers of AD patients. Iranian family caregivers consisted of persons provided care for the patient, was the family member, relatives, or friends of the patient (informal caregivers), and to participate in this study. They were selected using convenient methods through social groups and introducing people. The minimum sample size for factor analysis is 200 samples [30]; Therefore, a total of 435 family caregivers were recruited that were split randomly for assessing Exploratory Factor Analysis (EFA) and Confirmatory Factor Analysis (CFA) (210 for EFA and 225 for CFA).

### Measures

**Fig. 1:**
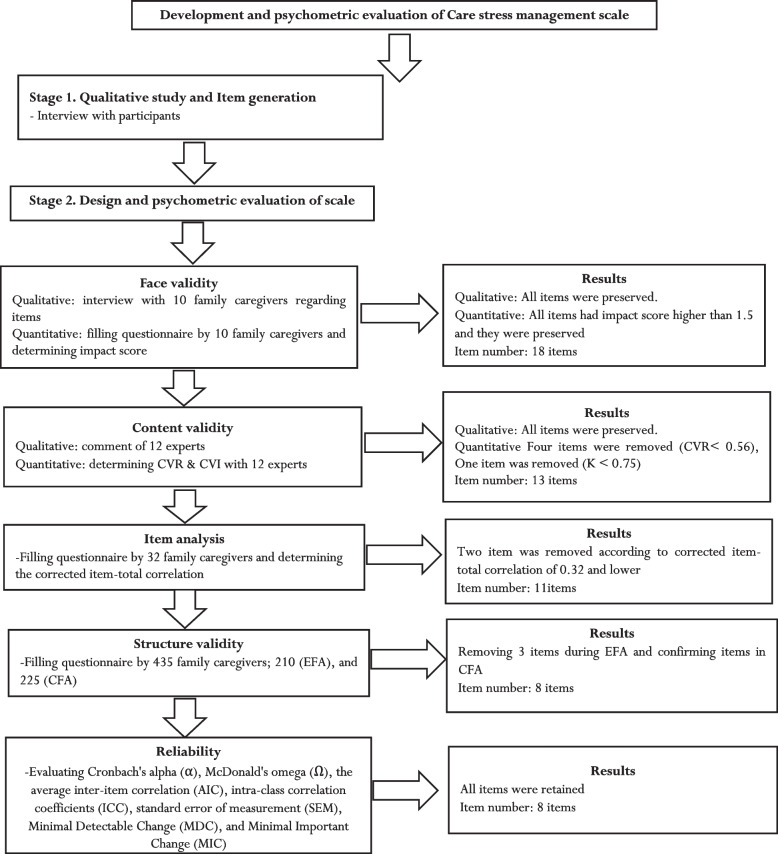
Production phases of Care stress management scale

An online questionnaire was designed. It consisted of two parts. The first part was demographic questionnaire such as age, sex, marital status, education level, employment and the second part was care stress management scaled designed for this study with 18 items and five-point Likert response options (1 = never, 2 = rarely, 3 = sometimes, 4 = often, 5 = always). The details of the production phases of CSMS (reduction and creation) are shown in Fig. 1. The questionnaire was created via Google form and its URL link was sent to participants by email or social networking applications such as Telegram channel or WhatsApp.

The construct validity was evaluated using the EFA and CFA. The EFA was performed using the maximum-likelihood method with Promax rotation. The sample adequacy and suitability were checked via the Kaiser–Meyer–Olkin (KMO) and Bartlett’s tests. A KMO values of higher than 0.9 was interpreted as excellent [31]. Also, the factors were extracted based on Horn’s parallel analysis and Exploratory Graph Analysis [32]. EGA has created similar or better accuracy was achieved by identifying unique factors than other more common factor reduction approaches and also EGA has been effective at replicating factor analytic findings as well as discovering new factor of constructs [33]. EGA is part of network psychometrics and focuses on estimating undirected network models [34]. In network psychometry, nodes represent psychological variables (e.g. questionnaire items) and the connections (i.e. edges) between the nodes represent the statistical relationships that need to be estimated [35]. When analyzing data generated by psychological tools, we may want to know whether the nodes are interconnected and form clusters that represent latent variables. If the latent variable model is the true underlying causal model, the indicators in the network model are expected to form strongly connected clusters for each latent variable [32]. As well an item of each latent factor was determined by accounting factor loading via the following formula: CV = 5.152 ÷ √ (n – 2), where CV was the number of extractable factors and “N” was the sample size [36]. Factor loading of almost 0.3 was acceptable. In the next step, CFA was used to confirm the factor structure determined by EFA. For this purpose, the CFA was checked using the maximum-likelihood method and the most common goodness-of-fit indices such as Chi-square (χ2) test, Chi-square/degree of freedom ratio (χ2/df) < 4, comparative fit index (CFI) > .90, incremental fit index (IFI) > .90, normed fit index (NFI) > .90, Tucker–Lewis index (TLI) > .90, relative fit index (RFI) > .90, root mean square error of approximation (RMSEA) < .08, Parsimonious Normed Fit Index (PNFI) > 0.50, Parsimonious Comparative Fit Index (PCFI) > 0.50 [37].

### Convergent and discriminant validity

The average variance extracted (AVE), maximum shared squared variance (MSV), and composite reliability (CR) was used to determine the convergent and discriminant validity; so that the AVE > 0.5 and CR greater than AVE were considered as criteria for the existence of the convergent validity and MSV less than AVE was considered as a criterion for the existence of the discriminant validity [38]. Furthermore, a new approach of Heterotrait - Monotrait Ratio (HTMT) criteria developed by Heseler was used for evaluating discriminant validity. The value of less than 0.85 was considered as the existence of discriminant validity [38].

### Reliability

The internal consistency of CSMS was evaluated using Cronbach’s alpha (α), McDonald’s omega (Ω), and the average inter-item correlation (AIC). Internal consistency was acceptable if Coefficient’s α and Ω values were > 0.7, AIC was 0.2 to 0.4 [39]. In the structural education model also CR and maximum reliability (MaxH) > 0.7 were considered as criteria to evaluate the reliability [39].

The stability of the CSMS was assessed by evaluating the intra-class correlation coefficients (ICC) using a two-way random effect model with the test-retest method and two-week interval in 25 family caregivers [40]. The acceptable value of ICC is more than 0.8 [41].

The absolute reliability was measured using the standard error of measurement (SEM) via the following formula: $\left(\textrm{SEM}={\textrm{SD}}_{\textrm{Pooled}}\times \sqrt{1- ICC}\right)$ [41].

The responsiveness was assessed by counting the Minimal Detectable Change (MDC) via the formula: MDC95 = SEM × √2 × 1.96 and Minimal important change (MIC) via the formula: MIC = 0.5 × SD of the Δ score respectively. It is noteworthy the interpretation of MIC needs to LOA that was evaluated using the following formula: LOA = d ± 1.96 × SD difference. Finally, interpretability was assessed by evaluating ceiling and floor effect and MDC [41].

### Multivariate normality and outliers

In the current study, univariate outliers were evaluated using distribution charts and multivariate outliers were evaluated through Mahalanobis distance *p* < .001. Furthermore, univariate normality distribution was checked by skewness (±3) and kurtosis (±7), and multivariate normality distribution was checked by Mardia’s coefficient > 8 [41].

### Data analysis

Data were analyzed using SPSS/AMOS version 26 and JASP0.16.0.2.

### Ethical consideration

The protocol of this study was checked and confirmed by the Ethics Committee of the Mazandaran University of Medical Sciences (IR.MAZUMS.REC.1401.079). In this study, informed consent was obtained from all participants and from their legal guardian(s) (in illiterate participants). Also participants were assured that their information would remain confidential and no information indicating their identity such as name will be reported. They were written permission to record audio. In the data-gathering stage, the necessary study information such as the purpose of the study, number of questions, the confidentiality of information, researcher profile, and ethics code of study were mentioned on the first page. Also, the questions were not displayed until the participants had read this information and were not satisfied to complete the scale by clicking on the “next button”.

## Results

### Item generation

From the interview with participants, the item pool with 275 items was generated using initial codes. Out of which 18 items were selected as items of the CSMS with a five-point Likert response (1 = never, 2 = rarely, 3 = sometimes, 4 = often, 5 = always).

### Item reduction

In the content validity step, based on results of CVR and Modified Kappa (K) five items were removed, and the total number of the CSMS was reduced from 18 to 13 items. During the item analysis step, two items were also removed and the final CSMS with 11 items was entered into the factor analysis step.

### Demographic profile of participants

In the construct step, a total of 435 family caregivers participated in this study. The mean ages were 50.26 ± 13.24 years. More of participants were women (50.6%). Most of them were married (68.7%) and 52.9% of them were daughters of patients. Table 1 shows the details of the demographic profile of participants.

**Table 1:** Demographic characteristics of participants (*n* = 435)

Variables	N (%)
Age	50.26 ± 13.24
Gender	
Female	220 (50.6)
Male	215 (49.4)
Marital status	
Single	92 (21.1)
Married	299 (68.7)
Divorced	14 (3.2)
Widow	30 (6.9)
Education level	
Illiterate	11 (2.5)
Less than diploma	30 (6.9)
Diploma	200 (46)
Academic	194 (44.6)
Employment	
Unemployed	42 (9.7)
Employed	161 (37)
Housewife	146 (33.6)
Retired	24 (5.5)
Free	62 (14.3)
Lifestyle	
Independent	262 (60.2)
With patients	173 (39.8)
Relationship with the patient	
Daughter	230 (52.9)
Son	57 (13.1)
Wife/midwife	57 (13.1)
Friend	34 (7.8)
Relative	57 (13.1)
Average hours of care per day (hour)	7.51 ± 5.51
Duration of the disease (year)	4.65 ± 2.52

### Construct validity

**Fig. 2:**
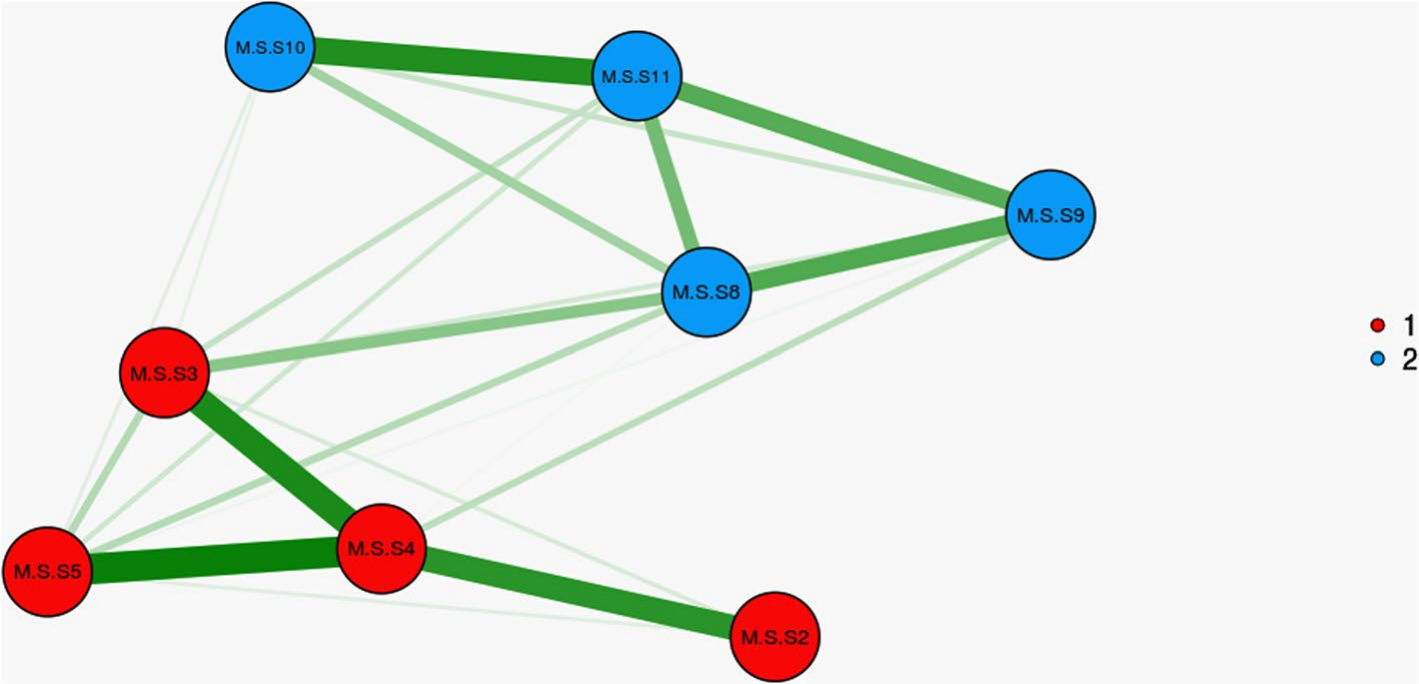
Exploratory Graph Analysis

**Fig. 3:**
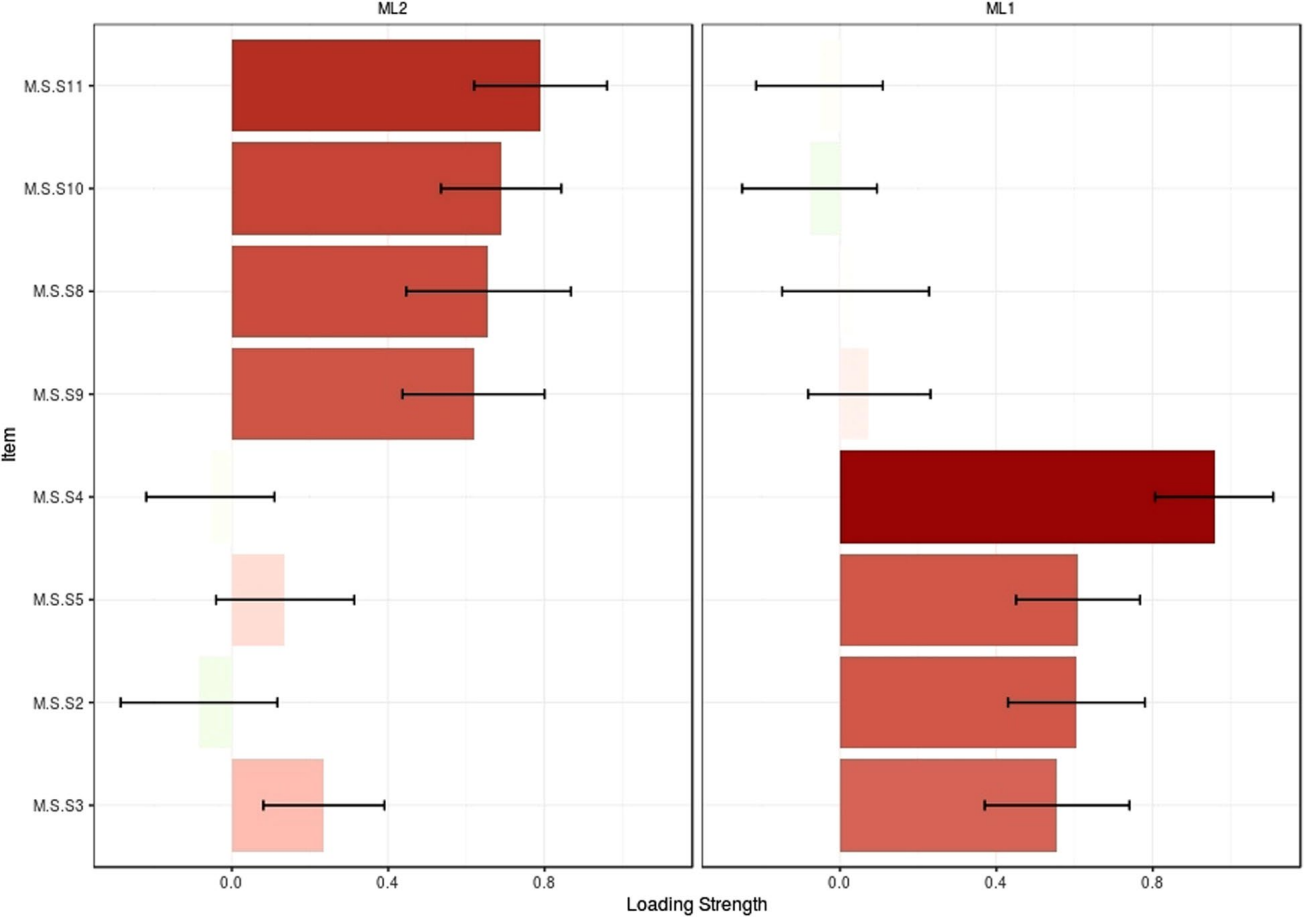
Loading strength of items in factors

The results of KMO (0.837) and Bartlett’s value 489.010 (*p* < .001) showed the sample was adequate and suitable. The EGA and parallel analyses revealed two factors. After Promax rotation in EFA, three items were removed and the total number of CSMS was removed to eight items that were classified into two factors namely “Emotional-focused coping” with four items and “Problem-focused coping” with four items. These two factors explained 51.00% of the total variance of care stress management concept in family caregivers of AD patients. Of this total variance, 26.57% was explained by the first factor and 24.43% was explained by the second factor. Table 2 and Figs. 2 and 3 show the details of factor analysis results.

**Table 2:** The result of EFA on the two factors of CSMS (*n* = 210)

Factors	Q_n_. Item	Factor loading	h^2a^	Eigenvalue	%Variance
Emotional-focused coping	11. By doing my favorite activities (eating, shopping, going to the movies, cooking, reading books, going to parties), I try to reduce my stress.	0.784	0.576	2.126	26.57
	10. By exercising, I try to reduce my stress.	0.689	0.421		
	8. To relieve stress, I engage in other activities.	0.654	0.458		
	9. By resting, I try to reduce my stress.	0.609	0.433		
Problem-focused coping	4. By focusing on problem solving, I reduce my stress.	0.961	0.845	1.953	24.43
	5. I try to reduce my stress by planning for daily activities	0.605	0.471		
	2. I try to calm my mind by realizing that my patient is unable to do own thing.	0.600	0.296		
	3. I try to control my stress by distracting myself in times of distress.	0.551	0.497		

^a^h^2^: Communalities

**Fig. 4:**
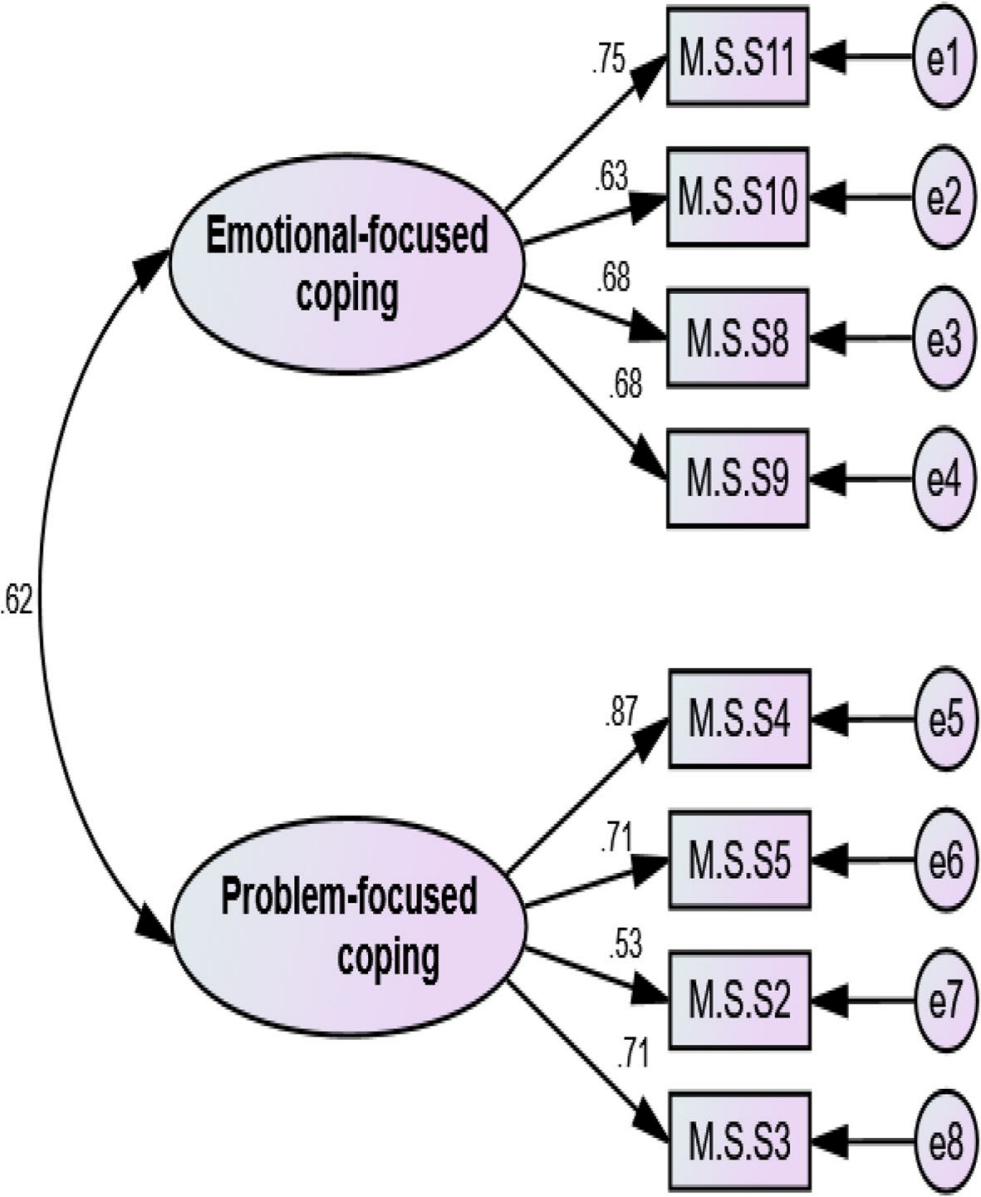
The CFA results of care stress management scale (*n* = 225)

During the CFA, the model extracted in EFA was tested. The results of the model fit indices showed that the model is fit and acceptable. Details of model fit indices have been shown in Table 3 and Fig. 4.

**Table 3:** Fit indices of the CFA Model after Structure Modification of the CSMS (*n* = 225)

Indices	χ^2^	df	*P* value	CMIN/DF	RMSEA	PNFI	NFI	RFI	PCFI	TLI	IFI	CFI
CFA Model	28.188	19	< .080	1.484	.052	0.640	0.943	0.917	0.665	.971	.981	.980

*DF* Degree of freedom, *PCFI* Parsimonious Comparative Fit Index, *PNFI* Parsimonious Normed Fit Index, *CMIN/DF* Minimum Discrepancy Function divided by Degrees of Freedom, *RMSEA* Root Mean Square Error of Approximation, *TLI* Tuker-Lewis Index, *CFI* Comparative Fit Index, *IFI* Incremental Fit Index

Fitness indexes: PNFI, PCFI (> 0.5); TLI, IFI, CFI, NFI, RFI (> 0.9), RMSEA (<0.08), CMIN/DF (<3 good, <5 acceptable)

Based on AVE, MSV, and CR results the first factor had the convergent and discriminant validity but the second factor did not have these validities. Also, the results of HTMT confirmed the absence of discriminant validity for the two factors of this scale (0.76). A detail of this validity has been shown in Table 4.

**Table 4:** The indices of the convergent, discriminant validity, and internal consistency of CSMS (*n* = 225)

	CR	AVE	MSV	MaxR (H)	Alpha	Omega	AIC
Emotional-focused coping	0.848	0.537	0.513	0.905	0.774	0.778	0.465
Problem-focused coping	0.745	0.372	0.513	0.758	0.791	0.802	0.487

DF: *CR* Composite reliability, *AVE* Average Variance Extracted, *MSV* Maximum Shared Squared Variance, *AIC* Average inter-item Correlation

Based on the results of Cronbach’s alpha, McDonald’s omega, and AIC, two factors of this scale have internal consistency (Cronbach’s alpha, McDonald’s omega > 0.7, AIC ≥ 0.4, and MaxR > 0.7). The details have been shown in Table 4. The results of ICC (0.844, CI 95: 0.531 to 0.948) showed the stability was strong (see Table 5). The absolute reliability based on SEM results was 1.65. Therefore, the scale score by repeat test for each person varies to the amount ± 1.65. It is noteworthy that based on the results of MDC, MIC, LOA, and ceiling and floor effect (items were free of these effects) this scale has responsiveness and interpretability. The details have been shown in Table 5.

**Table 5:** The results of stability, SEM, Responsiveness, and Interpretability

	ICC	SD	Mean	SEM	MDC95	MIC	LOA
**Scale**	0.844	4.13	30.30	1.65	4.57	2.06	22.20–114.58

## Discussion

Based on the results of this study, the CSMS is the valid and reliable scale to assess the care stress management concept in family caregivers of AD patients. It contains eight items that were divided into two factors: “Problem-focused coping” with four items and “Emotional-focused coping” with four items. The total variance of the care stress management concept in family caregivers of AD patients was explained by these two factors to a degree of 50.98%, with the first factor accounting for 26.57% of the variance and the second factor accounting for 24.43%. During the CFA, the model obtained with EFA was fit and acceptable. Based on the results of convergent and discriminant validity and HTMT, the first factor had convergent and discriminant validity but the second factor did not have these validities. Furthermore, this scale had excellent internal consistency and strong stability. Finally, the important and required domain of COSMIN (Consensus-based Standards for the selection of health measurement instruments) such as SEM, responsiveness, and interpretation [42] was evaluated for the CSMS. The SEM result was 1.65. Therefore, the scale score varies to the amount ± 1.65 by repeat test for each person. It is noteworthy that, since the SEM shows the accuracy of the measurement, the smaller value of SEM is important which was small for this scale. Finally, the CSMS has responsiveness and interpretability ability to show changes in a person’s situation over some time and the meaningfulness of changes.

Stress management is a strategy for coping with stress that can control stressful situations and reduce the resulting stress by making physical and emotional changes [43]. This coping strategy is categorized into two forms namely problem-focused coping and emotion-focused coping generally [44]. In this study, the two factors of CSMS were named “Emotional-focused coping” and “Problem-focused coping” that are discussed next.

The first factor was namely as “Emotional-focused coping” with four items which mean “trying to reduce the negative emotion caused by stress without trying to change a stressful situation that becomes the source of direct pressure” [45]. In CSMS, items related to this factor refer to mechanisms such as doing favorite activities (eating, shopping, going to the movies, cooking, reading books, going to parties), exercising, resting to manage stress, and adapting to a stressful situation. The Ways of Coping Checklist (WCC) developed by Folk man and Lazrus in 1980 year, is one of the more widely used measures for assessing coping strategies in a stressful situation. This scale has two factors i.e. problem-focused and emotional coping strategies. In WCCL-R, the sub-factors of emotional coping strategies includes seeking social support, self-indulgent escapism, seeking distance, negative avoidance, fanciful escapism [46]. Based on the findings of this study and the items of the first factor, family caregivers of AD patients try to reduce and regulate the stress of the situation by entertaining themselves with favorite activities and relaxing. It is noteworthy that the content of the items in CSMS is somewhat similar to the content of the subcategories of the WCC. In fact, these family caregivers also try to get away from stressful situations and their negative thoughts by doing their favorite activities and relaxing.

The second factor was named Problem-focused coping with four items that explain 24.43% of the total variance. Problem-focused coping involves all active efforts to manage stressful situations directly by correcting or eliminating sources of stress [47]. The content of items related to CSMS refers to mechanisms that family caregivers of AD patients use to correct or eliminate the source of stress and reduce the pressure. Based on items related to this factor, these family caregivers focus on problem-solving, planning for their daily activities, convincing themselves about the patient’s disability, and distracting themselves, try to manage stress and adapt to a stressful situation. Items of this factor are in line with the content of the subcategories of the Problem-focused coping factor of the WCC. Indeed, in the problem-focused coping factor of the WCC, pure problem focus, positive reinterpretation, directed problem-solving, self-control, responsibility, and spiritually based self-improvements were introduced as a mechanism to manage the stress and coping strategy [48].

### Study strength

In this study, we used the new methodological approaches such as Horn’s Parallel Analysis and Exploratory Graph Analysis for extracting factor structure. Furthermore, the important and required domains of COSMIN CHECKLIST such as accessing SEM, ICC, responsiveness, and interpretability were reported which increase the power and quality of the scale.

### Study limitation

Since the samples of this study were Iranian family caregivers of Alzheimer’s patients, the generalization of finding needs further testing in other caregivers and other cultures. Also, since data were gathered via an online questionnaire, and data were not collected physically and in face-to-face interviews, the accuracy of the answers may be questionable.

### Implication

As the number of Alzheimer’s patients is increasing and the care of this group of patients is a stressful situation, it is important to be aware of the stress management strategies used by these caregivers to reduce the negative effects of the situation. The CSMS with the fewer number of items, good explained variance, and being exclusive for this group is a useful scale for nurses, therapists, and researchers to assess the stress management strategy and make a plan to improve it.

## Conclusion

The finding of this study revealed that stress management strategy in family caregivers of patients with Alzheimer has two factors such as emotional-focused coping and problem-focused coping and CSMS is the valid and reliable scale with 8 items for assessing the stress management strategy in family caregivers of patients with Alzheimer.

## Data Availability

The datasets generated and analyzed during the current study are not publicly available due to our university rules, but are available from the corresponding author on reasonable request.
